# Supplementary material for: Plasma cell differentiation is regulated by the expression of histone variant H3.3
Source: Nat Commun. 2024 Jun 20;15:5004. doi: 10.1038/s41467-024-49375-x (PMC11190180; doi:10.1038/s41467-024-49375-x)
Supplement: Supplementary file 1 — Supplementary Information [file 41467_2024_49375_MOESM1_ESM.pdf]

Supplementary Information

Plasma cell differentiation is regulated by the expression of histone variant H3.3

Yuichi Saito et al.

Supplemental Figure 1

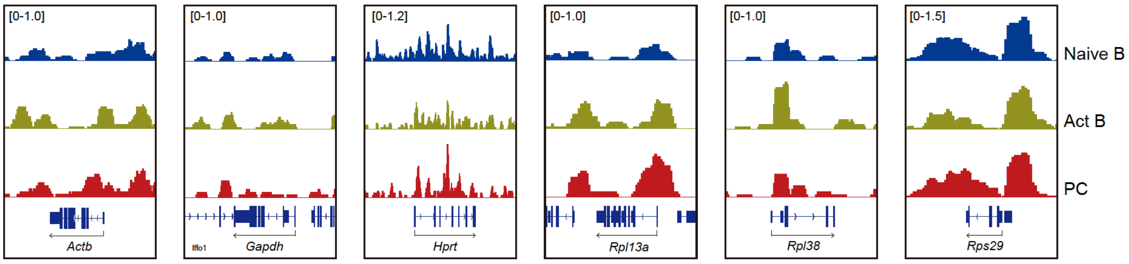

Supplementary Fig. 1.

Genome browser images showing representative housekeeping genes. Profiles of ChIL–seq data for H3.3 are shown in naive, Act B, and PC. All images were visualized by IGV (http://www.broadinstitute.org/igv/).

Supplementary Figure 2

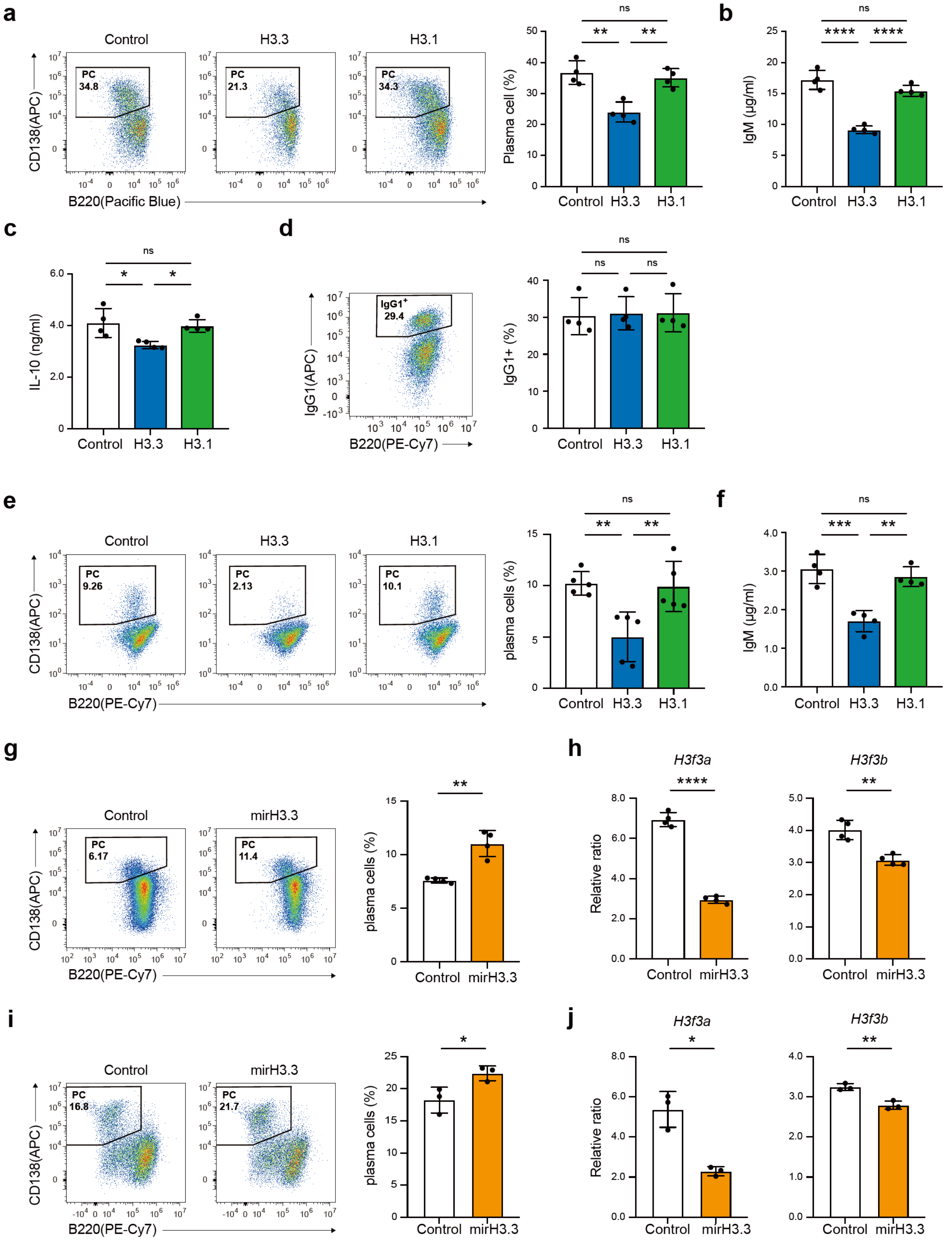

Supplementary Fig. 2.

a, Flow cytometry analysis of plasma cell differentiation in mCherry (control), mCherry-H3.3, or mCherry-H3.1 expressing B cells. **b,** The IgM antibody concentration in the culture supernatant of (a) was measured by ELISA. **c,** The IL-10 concentration in the culture supernatant, in which GFP (control), GFP-H3.3, or GFP-H3.1 expressing B cells induced IL-10 secretion by stimulation with LPS and anti-IgM, was measured by ELISA. **d**, Flow cytometry analysis of class-switch recombination to IgG1 in GFP (control), GFP-H3.3, or GFP-H3.1 expressing B cells. Examples of cells were pre-gated on live (Zombie $Aqua^-$) single lymphocytes $mCherry^+$ cells (a) and live (PI^-) single lymphocytes GFP^+ cells (d). **e,** Flow cytometry analysis of plasma cell differentiation in GFP (control), GFP-H3.3, or GFP-H3.1 expressing B cells on day 4 after the stimulation with anti-CD40 plus IL-2/IL-4/IL-5. Bars show the percentage of $CD138^+B220^{low}$ plasma cells among GFP^+ cells. **f,** The IgM antibody concentration in the culture supernatant of (**e**) was measured by ELISA. **g,** Representative flow cytometry plot of B cells retrovirally transduced with miR-H3.3 or control vector after stimulation with LPS for 2 days. Bars show the percentage of $CD138^+B220^{low}$ plasma cells (PCs) among GFP^+ cells. **h,** Quantitative RT-PCR of H3f3a and H3f3b mRNA in purified GFP^+ B cells was normalized to the expression of 18SrRNA in **g**. **i,** Representative flow cytometry plot of B cells retrovirally transduced with miR-H3.3 or control vector after stimulation with anti-CD40 plus IL-2/IL-4/IL-5 for 5 days. Bars show the percentage of PCs among GFP^+ cells. **j,** Quantitative RT-PCR of H3f3a and H3f3b mRNA in purified GFP^+ B cells was normalized to the expression of 18SrRNA on day 3 after stimulation with anti-CD40 plus IL-2/IL-4/IL-5. Data are representative of two independent experiments (a-d, f) and are pooled from two independent experiments (e). Data are presented as mean ± SD. *P < 0.05; **P < 0.01; ***P < 0.001; ****P < 0.0001; ns, not significant. The p values were obtained by one-way ANOVA with Tukey’s post hoc test (a-f) and by a two-tailed unpaired t-test with Welch’s correction (g-j).

Supplementary Figure 3

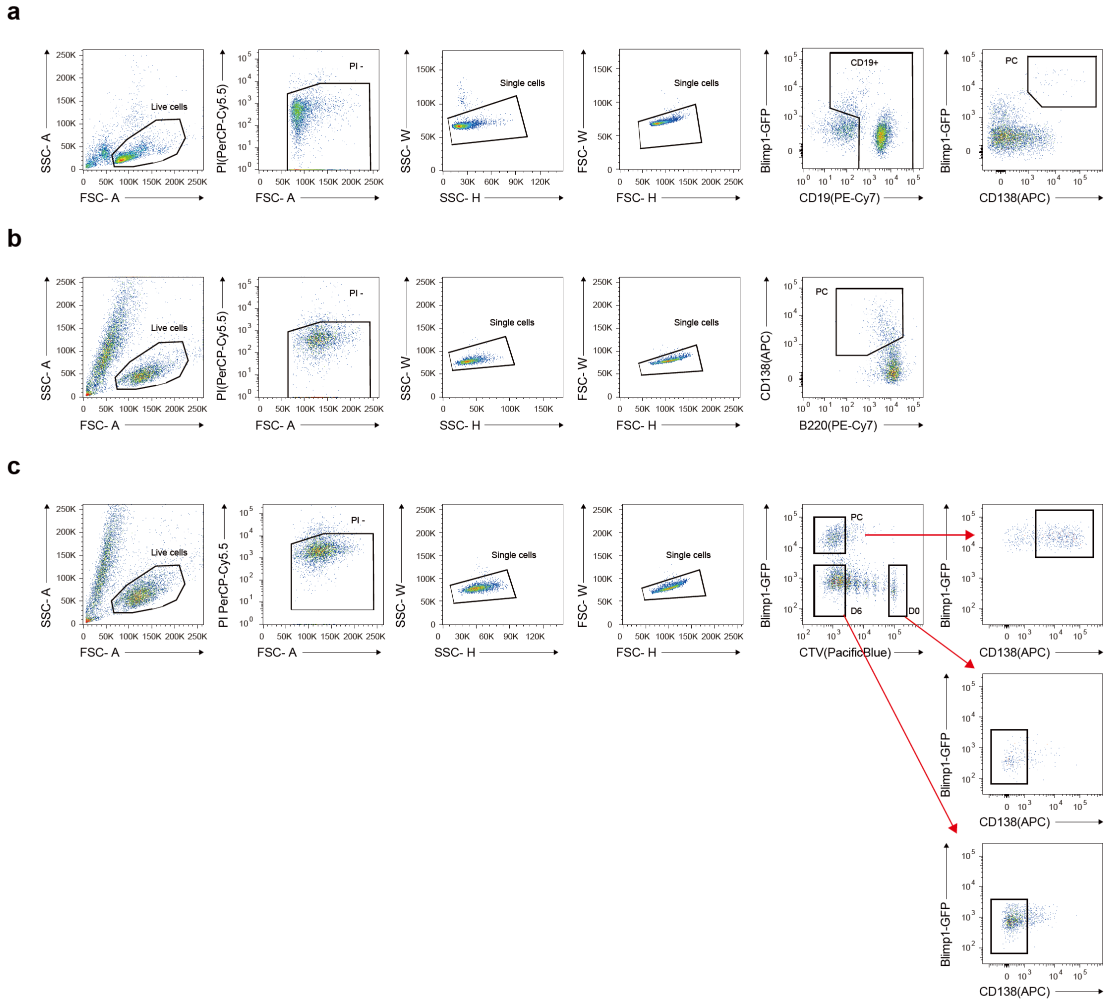

a, Gating strategy for Fig.1a. **b,** Gating strategy for Fig.1b. **c,** Gating strategy for Fig.1d.

Supplementary Figure 4

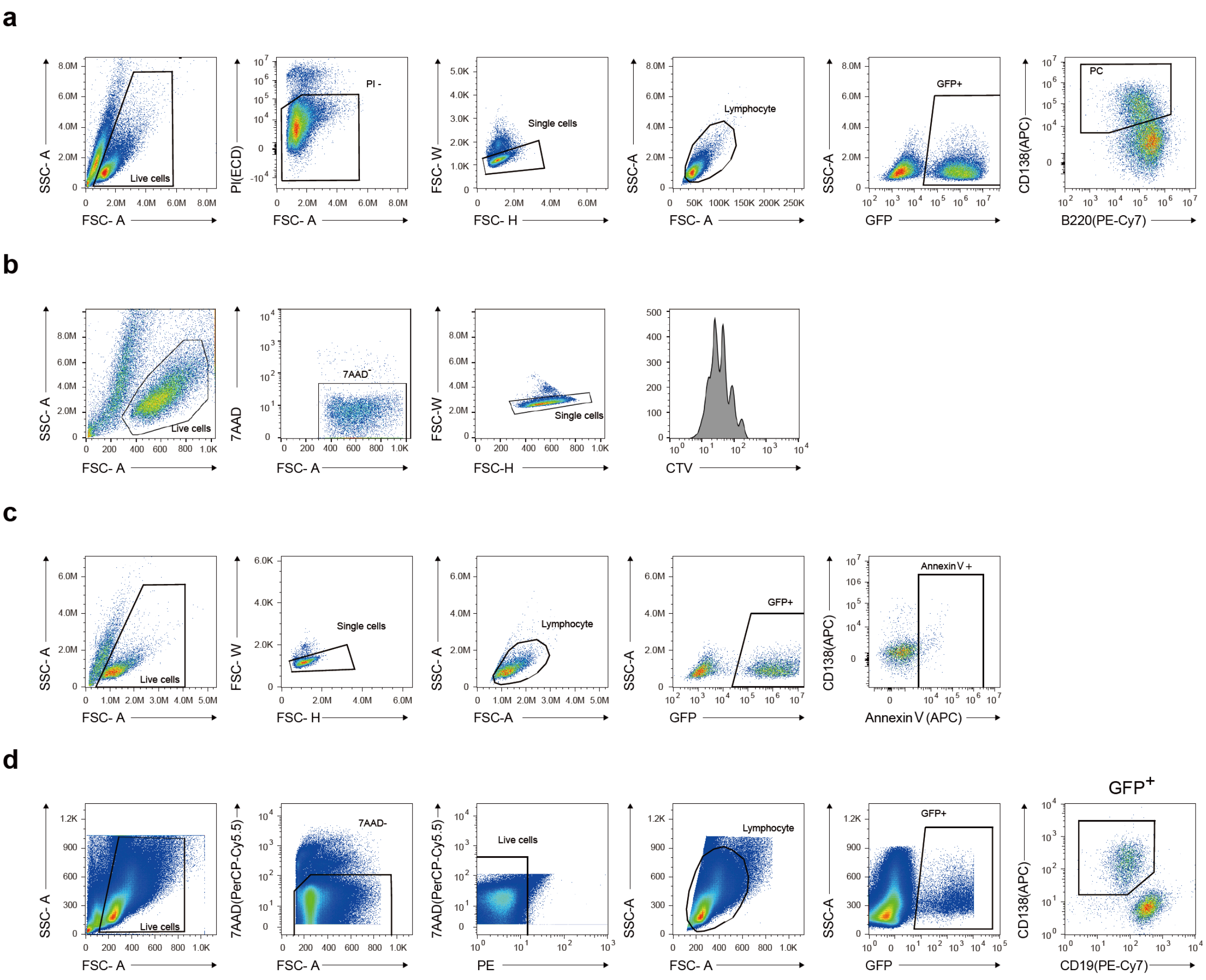

a, Gating strategy for Fig.3b. **b,** Gating strategy for Fig.3d. **c,** Gating strategy for Fig.3e. **d,** Gating strategy for Fig.3f.

Supplementary Figure 5

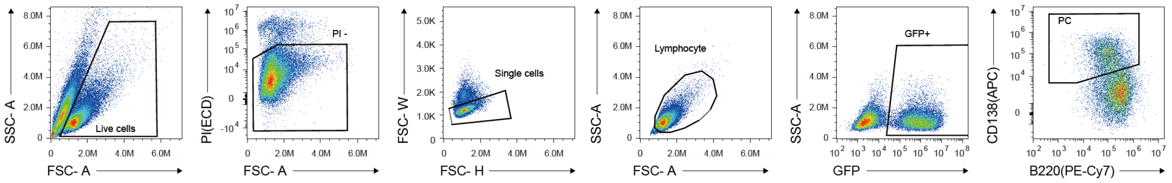

Gating strategy for Fig.4b.

Supplementary Figure 6

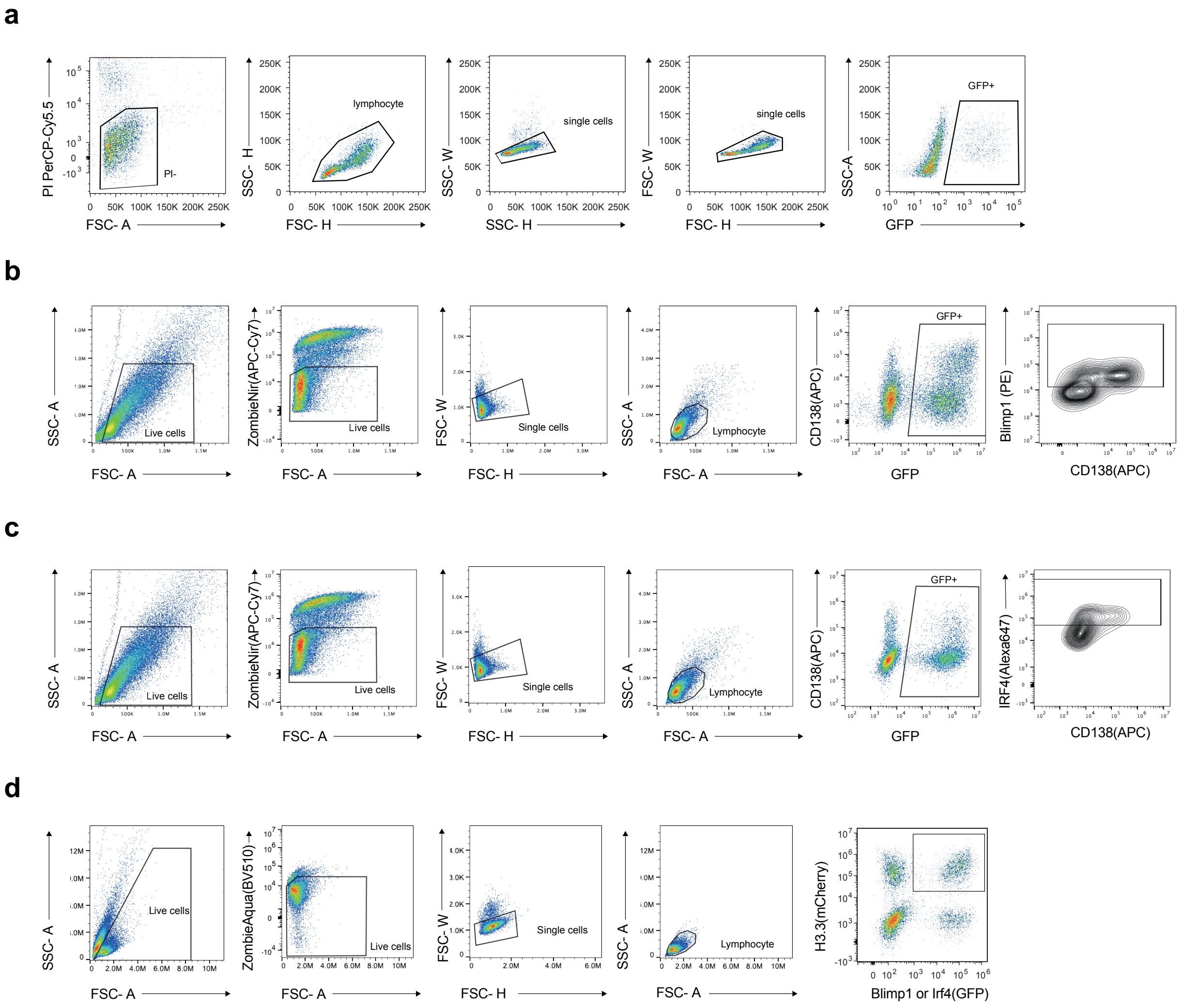

a, Sorting strategy for Fig.5a. **b,** Gating strategy for Fig.5b. **c,** Gating strategy for Fig.5c, **d,** Gating strategy for Fig.5d

Supplementary Figure 7

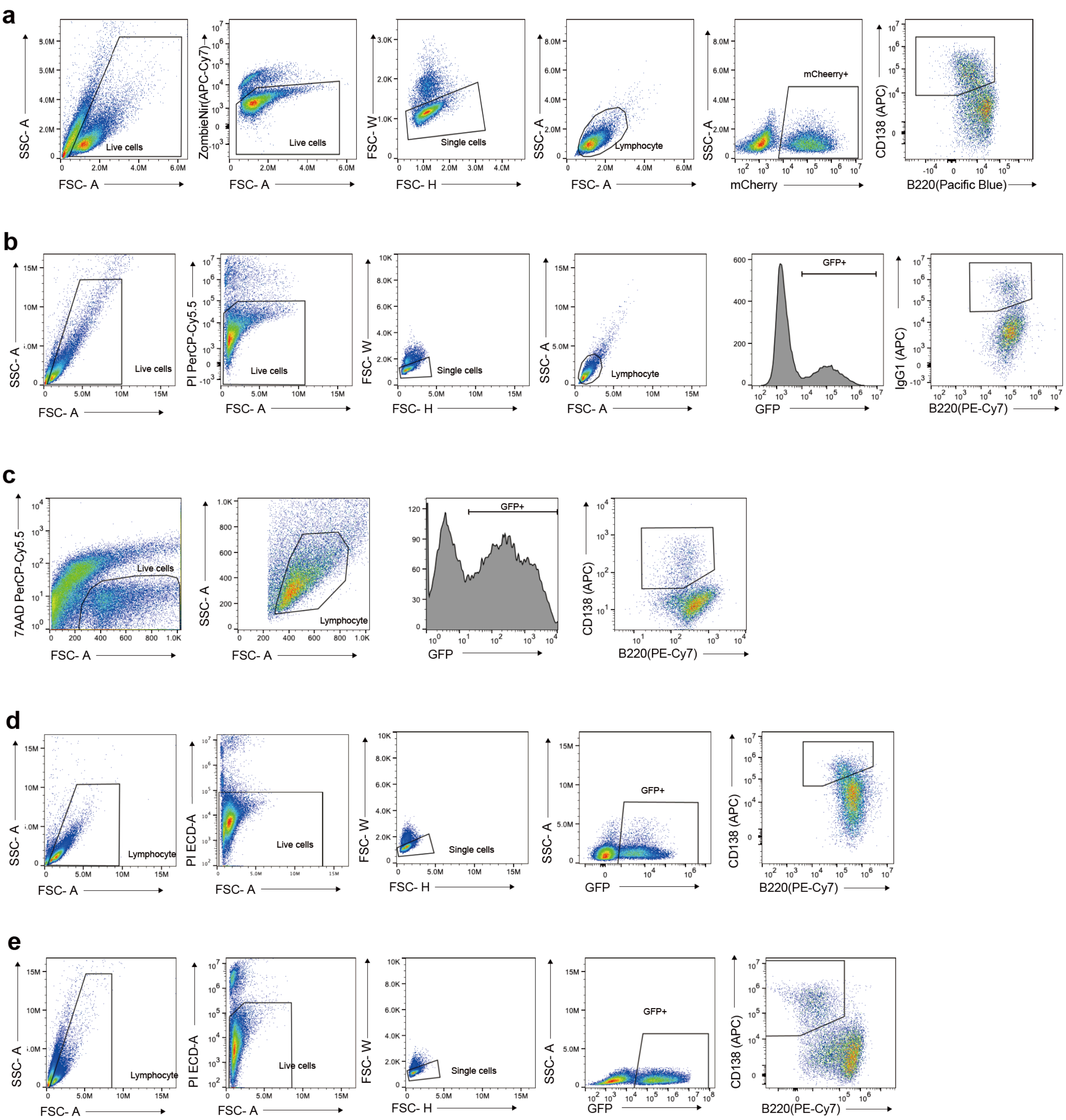

a, Sorting strategy for supplemental Fig.2a. **b,** Gating strategy for supplemental Fig.2d. **c,** Gating strategy for supplemental Fig.2e. **d,** Gating strategy for supplemental Fig.2g. **e,** Gating strategy for supplemental Fig.2i.

Supplementary Table 1.
Genomic feature distributions of H3.3 ChIL–seq reads.

Feature	Naive B (%)	Activated B (%)	PC (%)
Promoter	28.0035459	28.53644755	35.6392768
5' UTR	0.1633873	0.05650782	0.1407383
3' UTR	2.1553223	1.73290639	1.5697737
1st Exon	1.3766252	1.33735167	1.4290354
Other Exon	2.3239241	1.8835939	1.9053805
1st Intron	18.5774873	17.62102091	15.946736
Other Intron	26.055065	26.2949708	25.0514236
Downstream (<=300)	0.1268859	0.13185157	0.1515644
Distal Intergenic	21.2177571	22.40534941	18.1660712
