# Supplementary material for: Plasma cell differentiation is regulated by the expression of histone variant H3.3
Source: Nat Commun. 2024 Jun 20;15:5004. doi: 10.1038/s41467-024-49375-x (PMC11190180; doi:10.1038/s41467-024-49375-x)
Supplement: Supplementary file 3 — Reporting Summary [file 41467_2024_49375_MOESM3_ESM.pdf]

nature portfolio

Corresponding author(s): Yoshihiro Baba, Yasuyuki Ohkawa

Last updated by author(s): Apr 24, 2024

Reporting Summary

Nature Portfolio wishes to improve the reproducibility of the work that we publish. This form provides structure for consistency and transparency in reporting. For further information on Nature Portfolio policies, see our Editorial Policies and the Editorial Policy Checklist.

Statistics

For all statistical analyses, confirm that the following items are present in the figure legend, table legend, main text, or Methods section.

n/a	Confirmed	
☐	☒	The exact sample size (*n*) for each experimental group/condition, given as a discrete number and unit of measurement
☐	☒	A statement on whether measurements were taken from distinct samples or whether the same sample was measured repeatedly
☐	☒	The statistical test(s) used AND whether they are one- or two-sided<br>*Only common tests should be described solely by name; describe more complex techniques in the Methods section.*
☒	☐	A description of all covariates tested
☐	☒	A description of any assumptions or corrections, such as tests of normality and adjustment for multiple comparisons
☐	☒	A full description of the statistical parameters including central tendency (e.g. means) or other basic estimates (e.g. regression coefficient) AND variation (e.g. standard deviation) or associated estimates of uncertainty (e.g. confidence intervals)
☐	☒	For null hypothesis testing, the test statistic (e.g. *F*, *t*, *r*) with confidence intervals, effect sizes, degrees of freedom and *P* value noted<br>*Give P values as exact values whenever suitable.*
☒	☐	For Bayesian analysis, information on the choice of priors and Markov chain Monte Carlo settings
☒	☐	For hierarchical and complex designs, identification of the appropriate level for tests and full reporting of outcomes
☒	☐	Estimates of effect sizes (e.g. Cohen's *d*, Pearson's *r*), indicating how they were calculated

Our web collection on statistics for biologists contains articles on many of the points above.

Software and code

Policy information about availability of computer code

Data collection	The ChIL-seq and ATAC-seq libraries are sequenced using Illumina NovaSeq6000 platform. Flow cytometry data was collected using FACSChorus V.1.3, CytoExpert V.2.0. Western blot images were collected using Image Reader LAS3000 V.2.2. ELISA data was collected using Microplate Manager V.6. Real time PCR data was collected using LightCycler software.
Data analysis	Graph pad Prism 9, Flowjo V.10, trim_galore (version0.6.10), Bowtie2 (version 2.3.1), MACS2 (version 2.2.7.1), ChIPseeker(v1.33.1), R (version4.0.2) DESeq2 (version1.34.0) , deeptools (version 3.5.1), MEME-ChIP (version 5.4.1), Integrative Genomics Viewer (version 2.15.4).

For manuscripts utilizing custom algorithms or software that are central to the research but not yet described in published literature, software must be made available to editors and reviewers. We strongly encourage code deposition in a community repository (e.g. GitHub). See the Nature Portfolio guidelines for submitting code & software for further information.

Data

Policy information about availability of data

All manuscripts must include a data availability statement. This statement should provide the following information, where applicable:

- Accession codes, unique identifiers, or web links for publicly available datasets
- A description of any restrictions on data availability
- For clinical datasets or third party data, please ensure that the statement adheres to our policy

The ChIL-seq and ATAC-seq data including fastq, processed bigwig files and peak summit files (.bed) have been uploaded into the GEO GSE230496 (https://www.ncbi.nlm.nih.gov/geo/query/acc.cgi?acc=GSE230496). The reviewer token is ihavoykwpputrgz.

Research involving human participants, their data, or biological material

Policy information about studies with human participants or human data. See also policy information about sex, gender (identity/presentation), and sexual orientation and race, ethnicity and racism.

Reporting on sex and gender	n/a
Reporting on race, ethnicity, or other socially relevant groupings	n/a
Population characteristics	n/a
Recruitment	n/a
Ethics oversight	n/a

Note that full information on the approval of the study protocol must also be provided in the manuscript.

Field-specific reporting

Please select the one below that is the best fit for your research. If you are not sure, read the appropriate sections before making your selection.

☒ Life sciences ☐ Behavioural & social sciences ☐ Ecological, evolutionary & environmental sciences

For a reference copy of the document with all sections, see nature.com/documents/nr-reporting-summary-flat.pdf

Life sciences study design

All studies must disclose on these points even when the disclosure is negative.

Sample size	Sample size was determined to ensure reproducibility of experimental results with statistical significance, which is based on our previous studies and by availability of experimental animals as well.
Data exclusions	No data was excluded.
Replication	Biological and technical replicates were performed. Most of experiments were usually reproduced 2 to more than 3 independent times to ensure the reproducibility of findings.
Randomization	Randomization was not performed. All experimental groups were based on genotype of mice. Whenever possible, age/sex-matched mice were used.
Blinding	No experiment was performed blinded.

Reporting for specific materials, systems and methods

We require information from authors about some types of materials, experimental systems and methods used in many studies. Here, indicate whether each material, system or method listed is relevant to your study. If you are not sure if a list item applies to your research, read the appropriate section before selecting a response.

Materials & experimental systems

n/a	Involved in the study
☐	☒ Antibodies
☐	☒ Eukaryotic cell lines
☒	☐ Palaeontology and archaeology
☐	☒ Animals and other organisms
☒	☐ Clinical data
☒	☐ Dual use research of concern
☒	☐ Plants

Methods

n/a	Involved in the study
☒	☐ ChIP-seq
☐	☒ Flow cytometry
☒	☐ MRI-based neuroimaging

Antibodies

Antibodies used

For ChIL-seq, rat anti-H3.3 (4H2D7) homemade and rat anti-GFP (1A5) purchased from Bio Academia Co. Ltd. were used.
For flow cytometry analysis, the antibodies described below were used. Antibodies specific to B220 (RA3-6B2), CD19 (1:4005), CD138 (281-2), IgG1 (RMG1-1), TACI (8F10), IRF4 (IRF4.3E4) are from Biolegend. Antibodies specific to Blimp-1 (5E7) was purchased from BD Bioscience. Antibodies specific to GFP (polyclonal) was purchased from Invitrogen.
For western blot experiments, anti-H3.1/3.2(6G3C7) and anti-H3.3(6C4A3) antibodies(homemade) were used.
For ELISA experiments, anti-mouse IgM(polyclonal) ,HRP-conjugated anti-mouse IgM(polyclonal), mouse IgM(11E10) purchased from Southern Biotech were used.

ANTIGEN , DILUTION/AMOUNT, FLUOROCHROME(S)
Flow cytometry
B220, 1:400, Pacific Blue/PE-Cy7
CD19, 1:400, APC
CD138, 1:400, BV421 /APC
IgG1, 1:400, APC
TACI, 1:400, PE
IRF4, 1:400, Alexa647
Blimp-1, 1:200, PE

ChIL-sequence
H3.3, Final conc. per well: 0.2 μg/ml
GFP, Final conc. per well: 0.2 μg/ml

Western
H3.3, 1:5000
H3.1/H3.2 1:5000

Validation

For the ChIL-seq antibody including H3.3, GFP anti-bodies, previously articles have used in ChIP-seq or ChIP-qPCR.
All other antibodies used for flow cytometry and Western blot were passed manufacturer's quality controls and validated for indicated applications.

Flow cytometry
B220, Pacific Blue: https://www.biolegend.com/ja-jp/products/pacific-blue-anti-mouse-human-cd45r-b220-antibody-2857?GroupID=GROUP658
B220, PE-Cy7: https://www.biolegend.com/ja-jp/products/pe-cyanine7-anti-mouse-human-cd45r-b220-antibody-1930
CD19, APC
CD138, BV421 /APC: https://www.biolegend.com/ja-jp/products/apc-anti-mouse-cd19-antibody-1526
IgG1, APC: https://www.biolegend.com/ja-jp/products/apc-anti-mouse-igg1-7022
TACI, PE: https://www.biolegend.com/ja-jp/products/pe-anti-mouse-cd267-taci-antibody-5807
IRF4, Alexa647: https://www.bdbiosciences.com/ja-jp/products/reagents/flow-cytometry-reagents/research-reagents/single-color-antibodies-ruo/pe-mouse-anti-irf4.566649
Blimp-1, PE: https://www.bdbiosciences.com/ja-jp/products/reagents/flow-cytometry-reagents/research-reagents/single-color-antibodies-ruo/pe-rat-anti-mouse-blimp-1.564268
GFP, FITC : https://www.thermofisher.com/antibody/product/GFP-Antibody-Polyclonal/A-21311

ChIL-sequence and western blot
H3.3, H3.1
https://www.cosmobio.co.jp/product/detail/cac-20130109.asp?entry_id=10360

Eukaryotic cell lines

Policy information about cell lines and Sex and Gender in Research

Cell line source(s)

Plat-E Retroviral Packaging Cell Line, Ecotropic(Cell Biolab)

Authentication	Antibiotic selection by Puromycin and Blasticidin was performed.
Mycoplasma contamination	These lines were not tested for mycoplasma contamination.
Commonly misidentified lines (See ICLAC register)	No misidentified cell lines was used.

Animals and other research organisms

Policy information about studies involving animals; ARRIVE guidelines recommended for reporting animal research, and Sex and Gender in Research

Laboratory animals	Mus musculus; C57BL/6; Prdm1-GFP, μMT; Mice used are at the age of 8-12 week old.
Wild animals	No wild animals were used.
Reporting on sex	Sex does not affect the results of this study.
Field-collected samples	Study did not involve field-collected samples .
Ethics oversight	All mice were maintained in a specific pathogen-free animal facility following the ARRIVE guidelines and institutional guidelines and with protocols approved by the animal care and use committees at Kyushu University.

Note that full information on the approval of the study protocol must also be provided in the manuscript.

Plants

Seed stocks	n/a
Novel plant genotypes	n/a
Authentication	n/a

Flow Cytometry

Plots

Confirm that:

- [x] The axis labels state the marker and fluorochrome used (e.g. CD4-FITC).
- [x] The axis scales are clearly visible. Include numbers along axes only for bottom left plot of group (a 'group' is an analysis of identical markers).
- [x] All plots are contour plots with outliers or pseudocolor plots.
- [x] A numerical value for number of cells or percentage (with statistics) is provided.

Methodology

Sample preparation	Spleens were minced and single-cell suspension were passed through 80 micrometer strainers and red blood cell (RBC)s were lysed with ammonium chloride potassium buffer. For bone marrow (BM) cells, single-cell suspension was obtained by flashing BM cells out with the preparation medium from tibia and femur of mice. Then, RBCs were lysed as described.
Instrument	BD FACSMelody, Beckman Coulter Cytoflex S
Software	FACSChorus V.1.3, CytoExpert V.2.0, Flowjo V.10
Cell population abundance	The purity of sorted cells was> 95%, checked post sort.
Gating strategy	Before gating on the cells of interest shown in figure legend and method section, cells were gated by FSC-A/SSC-A to exclude debris and non-lymphocytes, then FSC-H/FSC-W to gate on singlet cells, followed by exclusion of dead cells using the viability dyes.

- [x] Tick this box to confirm that a figure exemplifying the gating strategy is provided in the Supplementary Information.
